# Single Low Dose Primaquine (0.25mg/kg) Does Not Cause Clinically Significant Haemolysis in G6PD Deficient Subjects

**DOI:** 10.1371/journal.pone.0151898

**Published:** 2016-03-24

**Authors:** Germana Bancone, Nongnud Chowwiwat, Raweewan Somsakchaicharoen, Lalita Poodpanya, Paw Khu Moo, Gornpan Gornsawun, Ladda Kajeechiwa, May Myo Thwin, Santisuk Rakthinthong, Suphak Nosten, Suradet Thinraow, Slight Naw Nyo, Clare L. Ling, Jacher Wiladphaingern, Naw Lily Kiricharoen, Kerryn A. Moore, Nicholas J. White, Francois Nosten

**Affiliations:** 1 Shoklo Malaria Research Unit, Mahidol-Oxford Tropical Medicine Research Unit, Faculty of Tropical Medicine, Mahidol University, Mae Sot, Thailand; 2 Mahidol-Oxford Tropical Medicine Research Unit, Faculty of Tropical Medicine, Mahidol University, Bangkok, Thailand; 3 Centre for Epidemiology and Biostatistics, Melbourne School of Population and Global Health, The University of Melbourne, Melbourne, Victoria, Australia; 4 Macfarlane Burnet Institute for Medical Research and Public Health, Melbourne, Victoria, Australia; 5 Centre for Tropical Medicine and Global Health, Nuffield Department of Medicine, University of Oxford, Oxford, United Kingdom; Centers for Disease Control and Prevention, UNITED STATES

## Abstract

**Background:**

Primaquine is the only drug consistently effective against mature gametocytes of *Plasmodium falciparum*. The transmission blocking dose of primaquine previously recommended was 0.75mg/kg (adult dose 45mg) but its deployment was limited because of concerns over haemolytic effects in patients with glucose-6-phosphate dehydrogenase (G6PD) deficiency. G6PD deficiency is an inherited X-linked enzymatic defect that affects an estimated 400 million people around the world with high frequencies (15–20%) in populations living in malarious areas. To reduce transmission in low transmission settings and facilitate elimination of *P*. *falciparum*, the World Health Organization now recommends adding a single dose of 0.25mg/kg (adult dose 15mg) to Artemisinin-based Combination Therapies (ACTs) without G6PD testing. Direct evidence of the safety of this low dose is lacking. Adverse events and haemoglobin variations after this treatment were assessed in both G6PD normal and deficient subjects in the context of targeted malaria elimination in a malaria endemic area on the North-Western Myanmar-Thailand border where prevalence of G6PD deficiency (Mahidol variant) approximates 15%.

**Methods and Findings:**

The tolerability and safety of primaquine (single dose 0.25 mg base/kg) combined with dihydroartemisinin-piperaquine (DHA-PPQ) given three times at monthly intervals was assessed in 819 subjects. Haemoglobin concentrations were estimated over the six months preceding the ACT + primaquine rounds of mass drug administration. G6PD deficiency was assessed with a phenotypic test and genotyping was performed in male subjects with deficient phenotypes and in all females. Fractional haemoglobin changes in relation to G6PD phenotype and genotype and primaquine round were assessed using linear mixed-effects models. No adverse events related to primaquine were reported during the trial. Mean fractional haemoglobin changes after each primaquine treatment in G6PD deficient subjects (-5.0%, -4.2% and -4.7%) were greater than in G6PD normal subjects (0.3%, -0.8 and -1.7%) but were clinically insignificant. Fractional drops in haemoglobin concentration larger than 25% following single dose primaquine were observed in 1.8% of the population but were asymptomatic.

**Conclusions:**

The single low dose (0.25mg/kg) of primaquine is clinically well tolerated and can be used safely without prior G6PD testing in populations with high prevalence of G6PD deficiency. The present evidence supports a broader use of low dose primaquine without G6PD testing for the treatment and elimination of falciparum malaria.

**Trial Registration:**

ClinicalTrials.gov NCT01872702

## Introduction

Artemisinin resistance in *Plasmodium falciparum* is spreading in South East Asia, followed by combination partner drug resistance [1]. Elimination of falciparum malaria in this region of low seasonal unstable transmission is considered the best option to contain this serious global threat [2, 3]. All possible measures to reduce malaria transmission in this area should be taken. Primaquine is the only available drug that reliably kills the mature circulating stages of *P*. *falciparum* gametocytes thereby preventing transmission to the mosquito vector. The World Health Organization (WHO) now recommends adding a single low dose of primaquine (0.25mg base/kg) to artemisinin combination treatment of *P*. *falciparum* malaria without prior testing for G6PD deficiency to reduce malaria transmission further in areas of low transmission and in the context of elimination [4, 5]. Although there is substantial indirect evidence that this small dose of primaquine is well tolerated in G6PD deficient patients, direct evidence is lacking.

Along the Thai-Myanmar border, malaria is caused by both *P*. *falciparum* and *Plasmodium vivax*. Malaria morbidity, mortality, and prevalence have declined over recent years, but resistance to artemisinins, followed by declining efficacy of the combination partner drugs, has emerged threatening these recent gains [6–8]. In the Karen and Burman populations living along the Thai-Myanmar border the prevalence of G6PD deficiency ranges from 9% to 18% in males and is caused mainly by the Mahidol mutation [9]. This variant is associated with a low to very low residual enzymatic activity in hemizygous males and homozygous females and causes haemolysis under oxidative stresses such as exposure to primaquine. As part of the Targeted Malaria Elimination (TME) study (ClinicalTrials.gov NCT01872702) we assessed the safety of repeated single low dose primaquine (0.25mg base/kg) administered with dihydroartemisinin-piperaquine (DHA-PPQ) in a population with high prevalence of G6PD deficiency.

## Materials and Methods

**Fig 1:**
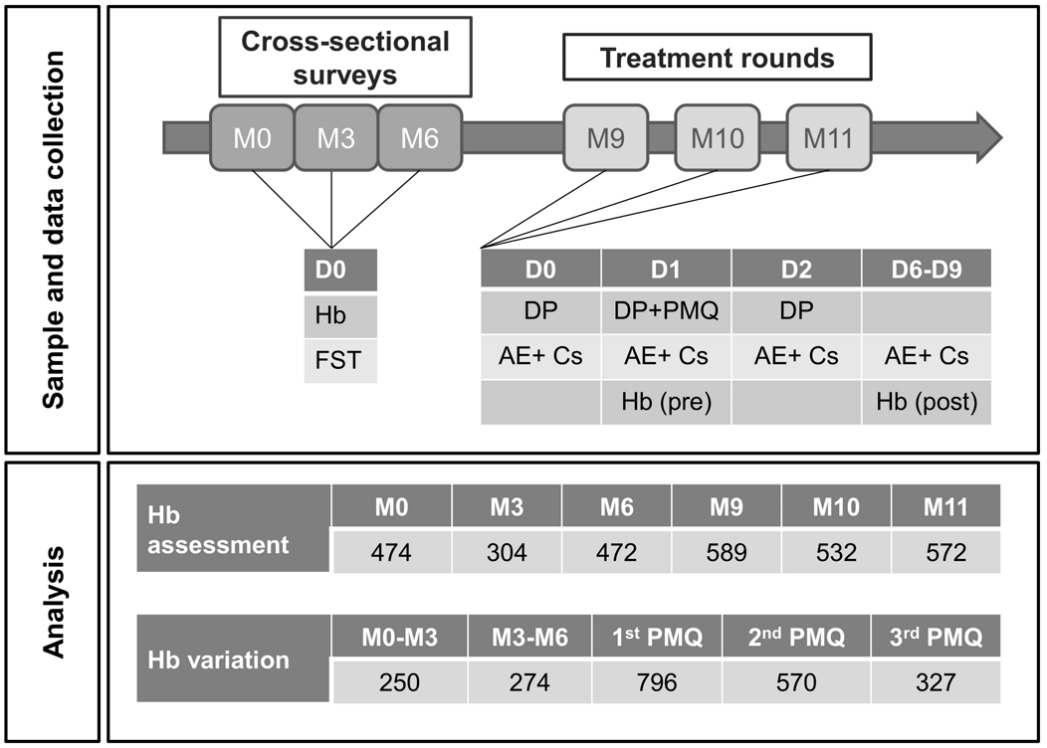
Study and analysis chart. Upper pane: outline of samples and data collection during surveys and drug administration rounds. Lower pane: haemoglobin data used for statistical analysis. M = months, D = day, Hb = haemoglobin concentration, FST = G6PD fluorescent spot test, DP = dihydroartemisinin-piperaquine dose, PMQ = primaquine dose, AE = Adverse Events, Cs = Complaints, Hb(pre) = Hb prior to primaquine treatment, (Hb)post = Hb after primaquine treatment

The TME study was conducted in four villages along the Thai-Myanmar border: TOT, KNH, HKT, and TPN during 24 months. In each village, exhaustive cross-sectional surveys were conducted every three months for the entire duration of the study and three monthly rounds of mass drug administration were given to all study participants. Each round consisted of three doses of DHA-PPQ (Sigma-Tau, Italy) and a single dose of 0.25 mg/kg of primaquine (Government Pharmaceutical Organization, Thailand). Only the villages of HKT and TPN, where additional haemoglobin levels were assessed at the time of treatment (at month 9, 10 and 11) and in the following week, are included in the following safety analysis (Fig 1).

### Cross-sectional surveys

At each survey, blood samples were obtained for malaria diagnosis and assessment of haemoglobin concentrations (Hb) using the HemoCue Hb201+ analyzer (Angelholm, Sweden). G6PD deficiency was determined by the Fluorescent Spot Test (FST, R&D Diagnostic, Greece) at recruitment and G6PD genotyping was performed subsequently in phenotypically deficient males and in all females. The most common local mutations: Mahidol (G487A), Chinese-4 (G329T), Canton (G1376T), and Viangchan (G871A) were characterised by PCR-RFLP methods [10]. When deficient subjects were found to have a mutation different from Mahidol, their female relatives were analyzed for the same mutation. A sub-group of subjects with G6PD deficient and intermediate phenotype by FST, for whom an additional fresh blood sample was available during the study follow-up, were further characterised by a G6PD quantitative spectrophotometric assay using Trinity kits (Trinity Biotech, Ireland) and by a flow-cytometric assay [11]. The results of the G6PD phenotypic tests were shared with the subjects and counselling was provided by the field nurse/medic when G6PD deficiency was detected. In addition, participants were given a card stating that they have “G6PD deficiency” and a list the drugs/foods to be avoided.

### Targeted malaria elimination (TME)

Male and female villagers older than six months were invited to receive the antimalarial regimen monthly for three months; pregnant women in the first trimester (confirmed by urine testing) and subjects with known history of allergy to any of the drugs were excluded. Pregnant women in the second and third trimesters were not given primaquine. Study participants received DHA-PPQ (7/55 mg/kg; Eurartesim®, Sigma-Tau, Italy) over three days and a single dose of primaquine (0.25mg base/kg; Government Pharmaceutical Organization, Thailand) at the second day of treatment. All medicines were given with a snack and under direct supervision. Adverse events and complaints (namely dizziness, itchiness and black urine) were recorded at each day of treatment and on day seven. Haemoglobin levels were assessed using the HemoCue Hb201+ analyzer the day of primaquine treatment and 5 to 8 days later.

### Statistical analysis

The present analysis included all subjects of villages HKT and TPN who received at least one round of targeted malaria chemoprevention. Fractional differences in haemoglobin concentrations before and after primaquine treatment were calculated as: (Hb post-treatment–Hb pre-treatment)/Hb pre-treatment. G6PD phenotype by FST was either deficient or normal (the latter included female subjects with intermediate activity); genotypes were grouped as wild type, heterozygote and hemizygote/homozygote. The effects of treatment round, G6PD phenotype and genotype, and quantitative G6PD activity on fractional haemoglobin changes were assessed using univariable and multivariable linear mixed-effects models with random-effects for village and individual. An interaction parameter between treatment round and G6PD group was included to assess the effect of G6PD phenotype and genotype for each primaquine round, and the effect of multiple primaquine rounds within G6PD phenotype and genotype groups, on fractional haemoglobin changes. For the analysis of natural variation of Hb levels over time, fractional changes in haemoglobin between two successive sampling (month 0–3 and month 3–6) were calculated for the same subjects who received primaquine. Data were obtained during three surveys carried out at the start (M0), three (M3) and six (M6) months.

Adverse events (AE) and reported complaints were compared among G6PD deficient and normal subjects using χ^2^ test. Post treatment fall in haemoglobin larger than 25% was defined as clinically important and described with respect to G6PD status, age and associated AE and complaints. Statistical analyses were performed using SPSS Version22 (IBM Corp., Armonk, NY, USA) and Stata Version 13 (StataCorp, College Station, Texas, USA).

The Oxford University Ethics Committee, the Tak Border Community Advisory Board and the village health committees approved the study. The purpose of the study was explained to all study subjects, or their parents/guardians, in their own language and an informed consent form was signed subsequently.

## Results

### Prevalence of G6PD deficiency (G6PD phenotypes and genotypes)

Of the 1400 subjects analyzed at any time during the whole study in the villages of HKT and TPN, 124 (8.9%) were G6PD deficient by FST and 1276 (91.1%) were normal (of whom 50 were women with an “Intermediate” phenotype), Table 1. Genotyping was performed on all the deficient subjects and on all female subjects. Males who had a normal phenotype were considered to have the wild type genotype. The most common mutations found among all mutated alleles were Mahidol (91.1%) followed by Chinese-4 (4.2%), Canton (3.7%) and Viangchan (0.9%). The complete results of genotyping are shown in S1 Table.

**Table 1:** G6PD phenotypic characterisation by FST.

Village	Sex	Deficient	Intermediate	Normal	Total
**HKT**	M	67 (13.3%)		435 (86.7%)	502
	F	16 (3.4%)	28 (5.9%)	428 (90.7%)	472
**TPN**	M	37 (16.4%)		188 (83.6%)	225
	F	4 (2.0%)	22 (10.9%)	175 (87.1%)	201
**Total**	M	104 (14.3%)		623 (85.7%)	727
	F	20 (3.0%)	50 (7.4%)	603 (89.6%)	673
	Total	124 (8.9%)	50 (3.6%)	1226 (87.6%)	1400

Quantitative assessment of enzymatic activity at steady state was performed in 109 subjects with deficient and intermediate phenotypes. Table 2 shows the results of enzymatic activity by genotype and allelic variant. Hemizygous males and homozygous females showed a low enzymatic activity: <15% in Mahidol and Canton and <30% for all the other mutations compared to the wild type population median; heterozygous women showed, as expected, an intermediate activity on average, with a broader distribution.

**Table 2:** Spectrophotometric enzymatic activity (IU/gHb) in G6PD hemizygous, homozygous and heterozygous subjects.

G6PD Genotype	Variant	N	Mean activity	SD	Median activity	% of Normal*
**Hemizygous males**	Mahidol	31	0.96	0.37	0.99	12.8
	Chinese-4	4	2.13	0.92	1.76	28.4
	Canton	3	0.29	0.09	0.24	3.9
	Viangchan	1	1.55	-	1.55	20.6
**Homozygous females**	Mahidol	5	1.03	0.47	0.88	13.7
**Heterozygous females**	Mahidol	61	5.43	2.01	5.12	72.3
	Canton	3	5.10	2.71	4.22	67.9
	Viangchan	1	5.49	-	5.49	73.1

*The normal median value of G6PD activity in the population, 7.51 IU/gHb, has been established previously [12].

### Haemoglobin changes after primaquine treatment by G6PD phenotype

A total of 1761 primaquine doses were administered during three month to 842 subjects; their mean (SD) age was 22.2 (18.4) years and they were 452 males and 390 females. For 13 subjects there was no available G6PD phenotype and for 55 primaquine doses the Hb was measured outside the 5–8 days post-treatment range so they were excluded from this analysis. Thus a total of 1693 primaquine doses given to 819 subjects who took one dose (252), two doses (260) and three doses (307) were included in the analysis. Baseline mean (SD) Hb concentrations were 10.8 (1.2) g/dL in children less than and equal to 5 years of age, 11.6 (1.2) g/dL in children 6–15 years of age, 13.4 (1.7) g/dL in adult males and 11.7 (1.6) g/dL in adult females. Baseline mean (SD) Hb concentrations were not different between G6PD deficient subjects and G6PD normal subjects overall [11.9 (1.7) g/dL *vs* 11.9 (1.7) g/dL] and at each treatment round [first round 12.0 (1.9) g/dL *vs* 12.0 (1.7) g/dL; second round 11.8 (1.5) g/dL *vs* 11.8 (1.6) g/dL; third round 11.6 (1.3) g/dL *vs* 11.9 (1.8) g/dL].

**Fig 2:**
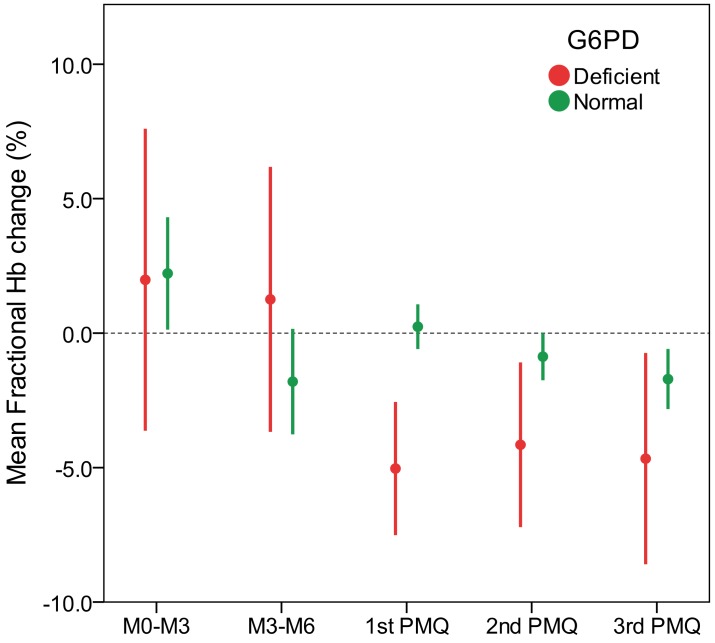
Mean and 95%CI fractional haemoglobin changes by G6PD phenotype. Only subjects who participated to the PMQ treatment rounds were included in the M0-M3 and M3-M6 analysis. In the PMQ rounds, data points of subjects with haemoglobin checked outside the 5–8 days post-treatment range were excluded from analysis. PMQ = primaquine, Hb = haemoglobin concentration.

Larger Hb falls were found in G6PD deficient subjects compared to G6PD normal, with mean fractional changes ranging from -5.0% [95% CI: -7.4, 2.6] to -4.2% [95% CI: -7.1, -1.2] in G6PD deficient subjects and -1.7% [95% CI: -3.0, -0.4] to +0.3% [95% CI: -0.6, 1.3] in G6PD normal subjects (Table 3 and Fig 2). The mixed-effects model used to analyze the data of repeated treatments showed that in the deficient subjects there was no change in haemolytic response in successive treatment rounds while in G6PD normal subjects there was a clear trend of increasing haemoglobin reductions across treatment rounds (Table 3); although changes were extremely small, G6PD normal subjects had significantly larger reductions in the second and third rounds compared to the first round.

**Table 3:** Analyses by linear mixed-effects modelling of mean fractional changes in haemoglobin by G6PD phenotype.

	Estimated fractional Hb change [95% CI]			Unadjusted mean differences between rounds [95% CI]	
	*1*^*st*^ *PMQ dose*	*2*^*nd*^ *PMQ dose*	*3*^*rd*^ *PMQ dose*	*2*^*nd*^ *vs 1*^*st*^ *PMQ dose*	*3*^*rd*^ *vs 1*^*st*^ *PMQ dose*
**Normal**	0.33[-0.64, 1.31]	-0.81[-1.88, 0.27]	-1.70[-3.01, -0.38]	-1.14[-2.31, 0.03]P = 0.056	-2.03[-3.43, -0.63],P = 0.004
**Deficient**	-5.00[-7.37, -2.64]	-4.17[-7.14, -1.21]	-4.77[-8.80, -0.74]	0.83[-2.86, 4.53],P = 0.658	0.24 [-4.36, 4.83],P = 0.920
** **	**Unadjusted mean differences between phenotypes [95% CI]**				
***Deficient vs Normal***	-5.34[-7.75, -2.93],P<0.001	-3.36[-6.40, -0.32],P = 0.030	-3.07[-7.23, 1.08],P = 0.147		
	**Overall adjusted mean difference between phenotypes [95% CI]** ^$^				
***Deficient vs Normal***	-4.32[-6.05, -2.60], P <0.001				

^$^ The overall adjusted mean difference is adjusted for round and age.

The normal fluctuations of Hb concentrations (before intervention) were measured in the same 819 subjects, in 250 subjects at the M0-M3 surveys and in 274 subjects at M3-M6 (Fig 2). The mean fractional Hb changes assessed prior to TME were +2.2% (95%CI: 0.2, 4.3) and -1.8% (95%CI: -3.7, 0.1) respectively in G6PD normal and 2.0% (95%CI: -3.6, 7.6) and +1.3% (95% CI: -3.6, 6.1) respectively in G6PD deficient. In both groups the individual variations were large.

### Haemoglobin reductions after primaquine treatment by G6PD genotype

Since there was no statistical difference in the reduction of haemoglobin between the various G6PD deficient genotypes (p_ANOVA_ = 0.7), the data from all hemizygous males for different variants were pooled. The same applied for heterozygous women carriers of different G6PD variants. The largest mean changes were found among the group of hemi and homozygous subjects; these ranged from -5.2% [95% CI: -7.6, -2.7] to -3.9% [95% CI: -6.9, -0.8]; haemoglobin reductions in heterozygous females ranged from -4.6% [95% CI: -7.0, -2.1] to -2.3% [95% CI: -5.5, 1.0] (Table 4). Wild type subjects had changes ranging from -1.7% [95% CI: -3.1, -0.2] to +0.8% [95% CI: -0.2, 1.9] (Table 4).

**Table 4:** Analyses by linear mixed-effects modelling of mean fractional changes in haemoglobin by G6PD genotype.

	Estimated fractional Hb change [95% CI]			Unadjusted mean differences between rounds [95% CI]	
	*1*^*st*^ *PMQ dose*	*2*^*nd*^ *PMQ dose*	*3*^*rd*^ *PMQ dose*	*2*^*nd*^ *vs 1*^*st*^ *PMQ dose*	*3*^*rd*^ *vs 1*^*st*^ *PMQ dose*
**wt**	0.84 [-0.23, 1.91]	-0.37 [-1.55, 0.81]	-1.67 [-3.10, -0.25]	-1.21 [-2.46, 0.04],P = 0.059	-2.51 [-4.00, -1.02],P = 0.001
**heterozygotes**	-3.18 [-5.29, -1.07]	-4.57 [-7.03, -2.11]	-2.28 [-5.54, 0.98]	-1.39 [-4.48, 1.70],P = 0.378	0.90 [-2.86, 4.66],P = 0.640
**hemi/homozygotes**	-5.16 [-7.65, -2.67]	-3.85 [-6.93, -0.77]	-4.04 [-8.30, 0.22]	1.31 [-2.53, 5.15],P = 0.503	1.13 [-3.71, 5.96],P = 0.648
	**Unadjusted mean differences between genotypes [95% CI]**				
***heterozygotes vs wt***	-4.02 [-6.17, -1.86],P<0.001	-4.20[-6.74, -1.65],P = 0.001	-0.61 [-4.03, 2.82],P = 0.728		
***hemi/homo zygotes vs wt***	-6.00 [-8.53, -3.48],P = <0.001	-3.48 [-6.63, -0.33],P = 0.031	-2.36[-6.75, 2.02],P = 0.291		
	**Overall adjusted mean difference between genotypes [95% CI]** ^$^				
***heterozygotes vs wt***	-3.45[-4.95, -1.96, P = <0.001				
***hemi/homo zygotes vs wt***	-4.55[-6.36, -2.75], P = <0.001				

^$^ The overall adjusted mean difference is adjusted for round and age.

G6PD wild type genotypes had increasing (very small) reductions over the three rounds of treatment (Table 4). Nonetheless even the largest mean Hb drop found in this group at the third round of treatment was very small and clinically insignificant.

### Haemoglobin falls and G6PD quantitative phenotypes

There was no association between the level of enzymatic activity and fractional haemoglobin changes (mean difference per one-unit increase in activity: 0.27%; 95% CI: -0.32, 0.86; p = 0.376) in 39 hemizygous, five homozygous, and 65 heterozygous subjects for whom a quantitative assessment of G6PD enzymatic activity was performed.

### Adverse Events and clinical assessment of haemolysis

Among all the 842 subjects who received the total 1761 primaquine doses, no serious adverse events related to primaquine were reported in the week following treatment. The only AE recorded happened in an adult woman who received only one treatment round; she received DHA-PPQ treatment on D0, D1 and D2. At D2 she was also given primaquine and her Hb level was assessed. At D9 (D7 post-primaquine) she had her Hb level measured again and she reported having passed black urine on D1. Her haemoglobin fell from 9.1g/dL at baseline to 6.8g/dL on D9. At the follow-up survey (two months after treatment) her haemoglobin concentration was 8.2g/dL. She was G6PD normal by FST but heterozygote for the Mahidol mutation; she was also a beta-thalassemia carrier and under treatment for tuberculosis and HIV. Further investigations of her G6PD phenotype confirmed that she had normal G6PD activity by spectrophotometric assay (8.01IU/gHb; population median = 7.51IU/gHb) and by the flow-cytometric assay 80% of her red cells had normal G6PD activity.

During the three rounds of treatment, complaints of dizziness were reported 79 times, eight among 150 G6PD deficient subjects (5.3%) with a median fractional haemoglobin difference of -9.0% and 71 among 1509 G6PD normal subjects (4.7%) with a median fractional haemoglobin difference of +2.1% (P = 0.73). Itchiness was reported in four subjects.

Out of the entire study population, 15 subjects (1.8%) had fractional haemoglobin falls greater than 25% at one treatment round (Table 5); four were G6PD phenotypically normal adults (including two heterozygous women), 11 were children <7years old of whom two were G6PD deficient (hemizygote for Mahidol mutation) and three were heterozygous Mahidol. Of these, eight occurred at first primaquine treatment while the remaining occurred at the second or third primaquine treatment. In addition, two adult women with normal FST and heterozygous genotype for Mahidol variant dropped Hb below 7g/dL. One woman dropped twice, from 7.4 to 6.1g/dL and from 8.3g/dL to 6.9 g/dL; she had HbH disease and her baseline Hb fluctuated between 7.3 and 9.6g/dL in M0-M6 period before treatment, suggesting that this was her normal level. The other woman dropped from 7.9g/dL to 6.9 g/dL and her Hb concentration varied from 10.6g/dL to 7.8g/dL in the months preceding the treatment rounds; Hb typing showed a slightly abnormal protein pattern compatible with a diagnosis of alpha-thalassemia. No subject dropped Hb below 5g/dL. None of these participants reported dizziness or black urine at any time during one week after treatment with the exclusion of the participant mentioned earlier.

**Table 5:** Demographic and haematologic parameters of subjects who experienced large haemoglobin drops (>25%) or fall below 7g/dL after primaquine dose.

**No**	**Village**	Age	sex	G6PD phenotype (FST)	G6PD phenotype (spectrophotometry)	Percentage of normal activity	G6PD*Mahidol	Hb (M0)	Hb (M3)	Hb (M6)	PMQ dose	Hb pre-PMQ (g/dL)	Hb post-PMQ (g/dL)	Fractional Hb drop (%)
**1**	TPN	3	F	Normal			Wild type	10.8		15.6	first	10.7	7.8	-27.1
**2**	TPN	35	M	Normal			Wild type	16.6		18.3	first	17.7	11.7	-33.9
**3**	TPN	1	M	Deficient			Hemizygote				second	13.6	10.1	-25.7
**4**	TPN	7	F	Normal	4.71	62.7	Heterozygote	11.8		10.5	first	10.9	8.2	-24.8
**5**	HKT	7	F	Normal			Heterozygote	12.9	12.5	10.7	first	16.1	10.7	-33.5
**6**	HKT	6	M	Normal			Wild type		11.5	11.1	first	11.3	8.4	-25.7
**7**	HKT	5	M	Normal			Wild type	11.3	11.4	10.6	third	17.5	10.2	-41.7
**8**	HKT	4	F	Normal	5.96	79.4	Heterozygote	10.2	11.2	9.8	second	11.6	8.7	-25.0
**9**	HKT	1	F	Normal			Wild type		10.5		second	12.1	9.1	-24.8
**10**	HKT	56	F	Intermediate	2.83	37.7	Heterozygote	11.0	11.4		second	10.7	8.0	-25.2
**11**	HKT	5	M	Normal			Wild type	11.5	12.7	10.7	first	11.8	8.0	-32.2
**12**	HKT	30	M	Normal			Wild type	14.2	12.9	13.9	second	14.7	10.8	-26.5
**13**	HKT	35	F	Normal	8.18	108.9	Heterozygote				first	9.1	6.8	-25.3
**14**	HKT	7	F	Normal			Wild type				second	12.2	9.1	-25.4
**15**	HKT	1	M	Deficient			Hemizygote				first	14.2	9.5	-33.1
**16**	HKT	21	F	Normal	6.94	92.4	Heterozygote	8.5	7.3	9.6	first	7.4	6.1	-17.6
**17**	HKT	21	F	Normal	6.94	92.4	Heterozygote	8.5	7.3	9.6	second	8.3	6.9	-16.9
**18**	HKT	35	F	Normal			Heterozygote	10.6		7.8	second	7.9	6.9	-12.7

## Discussion

This study was conducted to characterise the clinical tolerability and safety of the gametocytocidal 0.25mg base/kg dose of primaquine by quantifying drug-induced haemolysis in a large population of subjects with a known G6PD status. In 2012, when the WHO reviewed the safety of a single 0.75mg/kg dose of primaquine for blocking falciparum malaria transmission and recommended the lower dose of 0.25mg/kg, there were few data on haemoglobin falls after treatment with the single high (0.75mg/kg) dose and the limited characterisation of the G6PD status of the subjects involved in such studies was highlighted (summarized in S2 Table) [13].

Before this study, quantitative data evaluating the effect of single dose primaquine on haemoglobin levels could be found in only three studies, all performed in Africa where the relatively mild African A- genotype predominates. One of these studies investigated the use of two different low doses of primaquine, 0.1mg/kg and 0.4mg/kg, administered together with artemether-lumefantrine (AL) in malaria patients with a known G6PD status [14–16]. The average fractional falls observed in the G6PD normal group were 0.2% (AL alone), 2.2% (AL+0.1mg/kg primaquine) and 1.3% (AL+0.4mg/kg primaquine). In the group of G6PD hemi and homozygous subjects, the mean haemoglobin falls were 8.6% (AL alone), <0.1% (AL+0.1mg/kg primaquine) and 4.3% (AL+0.4mg/kg primaquine). The difference in haemoglobin falls between G6PD normal and hemi/homozygous was significant for the 0.4mg dose; however large variations in haemoglobin levels were seen also in the placebo group and the relative contributions of the drug treatment and malaria infection to the degree of haemoglobin reduction could not be assessed.

In this study using single 0.25mg base/kg doses of primaquine, we also observed greater falls in haemoglobin in the G6PD deficient subjects compared to G6PD normal subjects, with an overall mean difference after correction for age and multiple primaquine doses of -4.32% (95% CI-6.05, -2.60; P <0.001). However, the mean changes in the haemoglobin concentrations in both G6PD deficient (from -5.0% to -4.2%) and G6PD normal (from -1.7% to 0.3%) were small. Indeed, some large individual variations in haemoglobin levels were seen after primaquine treatment in both phenotypic groups, but were in magnitude not dissimilar from the haemoglobin fluctuations observed over time during the surveys preceding the treatments rounds (Fig 2).

Following treatment with an oxidative drug, the degree of haemolysis is expected to correlate with the number of red blood cells that are G6PD depleted; in subjects with clinically significant haemolysis, the erythropoietic response generates an increase in circulating reticulocytes with relatively high G6PD activity which renders the blood more resistant to the oxidative challenge of subsequent primaquine doses (a phenomenon called the “resistance phase”). In this study there was no correlation between the enzymatic activity analysed by quantitative spectrophotometry in steady state and the level of haemoglobin fall after treatment in subjects with intermediate and deficient G6PD activity. G6PD deficient subjects who received more than one round of primaquine treatment did not show any significant changes in the haemolytic response, possibly because the changes were so small and thus the power to show a significant change against a background of natural variation was limited. G6PD normal subjects showed increasing haemoglobin reductions over time with multiple treatments, but these were extremely small (maximum 2%) and of no clinical relevance.

In the past a higher single dose of primaquine (0.75 or 0.9 mg/ kg) was used in antimalarial trials in this area, and although haemoglobin levels were not analyzed, no serious adverse effects were reported [17]. As confirmation of this indirect evidence of safety obtained previously, the haemoglobin falls seen in both G6PD normal and deficient subjects in this study did not translate into clinical symptoms. No AE related to primaquine were reported and only 1.8% of participants experienced a large fractional Hb falls (>25%) with no reported complaints. In the entire treated population most of the reported complaints were of mild severity.

### Limitations of the study

One of the biggest limitations of the study is that the estimations of haemoglobin changes both in the surveys and in the treatment rounds showed substantial variability and so have large confidence intervals. Large intra-subjects Hb variations have been observed before where blood samples were taken at different time points in the same person or at the same time from fingers of left and right hands in adults [18] [19]. This limited the statistical power to assess the contribution of primaquine treatments to changes of Hb levels over the natural intra-subject Hb fluctuations. Additionally for the primaquine rounds the measurement process was carried out in the field and was technically challenging adding more imprecision in the assessment of Hb levels. These findings are limited to G6PD mutations found in this population and although G6PD*Mahidol is a severe variant with a residual enzymatic activity estimated to be 5–15% of normal, the safety data provided here might not apply to more severe and rare variants such as those causing chronic non-spherocytic haemolytic anaemia.

In conclusion, our haematological and clinical data provide evidence of the safety of single low dose of primaquine in populations with a high prevalence of G6PD deficiency and so support a broader use of low dose primaquine without G6PD testing for the treatment and elimination of falciparum malaria.

## Supporting Information

S1 Table G6PD genotypes according to FST phenotypes. (DOCX)

S2 Table Overview of mass drug administration studies (MDA) and clinical trials where single high and low doses of primaquine were used. (DOCX)

S3 Table Study dataset. (XLSX)
