# Supplementary material for: Single Low Dose Primaquine (0.25mg/kg) Does Not Cause Clinically Significant Haemolysis in G6PD Deficient Subjects
Source: PLoS One. 2016 Mar 24;11(3):e0151898. doi: 10.1371/journal.pone.0151898 (PMC4807095; doi:10.1371/journal.pone.0151898)
Supplement: S1 Table — (DOCX) [file pone.0151898.s001.docx]

***Table S1*. G6PD genotypes according to FST phenotypes**

|  | Genotype | FST | | | total |
| --- | --- | --- | --- | --- | --- |
|  |  | Deficient | Intermediate | Normal |  |
| HKT | Mahidol hemizygote | 44 | 0 | 1^#^ | 45 |
|  | Canton hemizygote | 4 | 0 | 0 | 4 |
|  | Chinese-4 hemizygote | 4 | 0 | 0 | 4 |
|  | Mahidol heterozygote | 5 | 19 | 39 | 63 |
|  | Canton heterozygote | 0 | 2 | 1 | 3 |
|  | Chinese-4 heterozygote | 0 | 1 | 0 | 1 |
|  | Mahidol homozygote | 4 | 0 | 0 | 4 |
|  | WT^@^ | 3* | 7 | 173 | 186 |
|  | Total | 64 | 29 | 214 | 310 |
| TPN | Mahidol hemizygote | 33 | 0 | 0 | 33 |
|  | Viangchan hemizygote | 1 | 0 | 0 | 1 |
|  | Canton hemizygote | 1 | 0 | 0 | 1 |
|  | Mahidol heterozygote | 3 | 18 | 23 | 44 |
|  | Viangchan heterozygote | 0 | 0 | 1 | 1 |
|  | Mahidol homozygote | 1 | 0 | 0 | 1 |
|  | WT^@^ | 2* | 7 | 114 | 130 |
|  | Total | 40 | 25 | 138 | 211 |

#possibly a laboratory error in FST interpretation

^@^Both females and males

* Deficient phenotypes associated with other mutations
