# Supplementary material for: Single Low Dose Primaquine (0.25mg/kg) Does Not Cause Clinically Significant Haemolysis in G6PD Deficient Subjects
Source: PLoS One. 2016 Mar 24;11(3):e0151898. doi: 10.1371/journal.pone.0151898 (PMC4807095; doi:10.1371/journal.pone.0151898)
Supplement: S2 Table — (DOCX) [file pone.0151898.s002.docx]

|  | Qualitative | | Quantitative | | | |
| --- | --- | --- | --- | --- | --- | --- |
| Type of study | MDA | Cohort-studies | Clinical trial | Clinical trial | Clinical trial | MDA |
| Place | Several countries | Several Countries | East-Africa + Congo | Tanzania | Uganda | Thai-Myanmar border |
| N Subjects included | >75000 | 3771 | 235 | 565 | 345 | 819 |
| Adults/Children | A+C | A+C | C | C | C | A+C |
| Healthy/ Malaria | H+M | H+M | M with anaemia | M | M | H |
| PMQ dose | 0.5-0.75mg/Kg | 0.5-0.67-0.75mg/Kg | 0.14-0.23mg/kg | 0.75mg/kg  (based on age) | Placebo- 0.1- 0.4- 0.75mg/Kg | 0.25mg/kg |
| PMQ regimen | Single | Single or weekly | Weekly | Single | Single | Single monthly |
| [Hb] pre-post PMQ | No | No | Yes | Yes | Yes | Yes |
| Δ[Hb] post PMQ | NA | NA | ±5% in 84.6% of children  5-10% in 3.1% of children | -0.5 g/dl in G6PD WT  -1.6 g/dl in G6PD heterozygotes  -2.5 g/dl in G6PD hemi/homozygotes | -0.2% (AL alone)  -2.2% (0.1mg/kg PMQ)  -1.3% (0.4mg/kg PMQ)  -3.3% (0.75mg/kg PMQ) in G6PD WT.  -8.6% (AL alone)  <0.1% (0.1mg/kg PMQ)  - 4.3% (0.4mg/kg PMQ)  -8.0% (0.75mg/kg PMQ) in G6PD hemi and homozygous. | From -1.7% to 0.3% in G6PD normal  From -5.0% to -4.2% in G6PD deficient |
| [Hb] overtime | No | No | No | No | No | Yes |
| G6PD deficiency present in population | Likely | Likely | Likely | Yes | Yes | Yes |
| G6PD phenotype | Not done | Not done | Not done | Not done | Qualitative | Qualitative and quantitative |
| G6PD genotype | Not done | Not done | Not done | G6PD*A- | G6PD*A- | G6PD*Mahidol, Chinese-4, Viangchan, Canton |
| Severe AEs (N) | 1 | 16 | None | 1 | None | None |
| Severe AEs (type) | Severe anaemia | Acute and severe haemolysis |  | Severe anaemia |  |  |
| Reference | Recht et al., 2014; Ch. 3.3-3.4 | Recht et al., 2014; Ch. 3.3-3.4 | Hodgkinson et al., 1961 | Eziefula et al., 2014a | Eziefula et al., 2014b | This study |

**Table S2. Overview of mass drug administration studies (MDA) and clinical trials where single high and low doses of primaquine were used.**
